# Estimation of brain network ictogenicity predicts outcome from epilepsy surgery

**DOI:** 10.1038/srep29215

**Published:** 2016-07-07

**Authors:** M. Goodfellow, C. Rummel, E. Abela, M. P. Richardson, K. Schindler, J. R. Terry

**Affiliations:** 1 College of Engineering, Mathematics and Physical Sciences, University of Exeter, Exeter, UK; 2 Centre for Biomedical Modelling and Analysis, University of Exeter, Exeter, UK; 3 EPSRC Centre for Predictive Modelling in Healthcare, University of Exeter, Exeter, UK; 4 Support Center for Advanced Neuroimaging (SCAN), University Institute for Diagnostic and Interventional Neuroradiology, University of Bern, Switzerland; 5 Institute of Psychiatry, Psychology and Neuroscience, King’s College London, London, UK; 6 Department of Neurology, University of Bern, Switzerland

## Abstract

Surgery is a valuable option for pharmacologically intractable epilepsy. However, significant post-operative improvements are not always attained. This is due in part to our incomplete understanding of the seizure generating (ictogenic) capabilities of brain networks. Here we introduce an *in silico*, model-based framework to study the effects of surgery within ictogenic brain networks. We find that factors conventionally determining the region of tissue to resect, such as the location of focal brain lesions or the presence of epileptiform rhythms, do not necessarily predict the best resection strategy. We validate our framework by analysing electrocorticogram (ECoG) recordings from patients who have undergone epilepsy surgery. We find that when post-operative outcome is good, model predictions for optimal strategies align better with the actual surgery undertaken than when post-operative outcome is poor. Crucially, this allows the prediction of optimal surgical strategies and the provision of quantitative prognoses for patients undergoing epilepsy surgery.

Surgery has been shown to be an effective treatment for seizures in pharmacologically intractable epilepsy^1^,^2^,^3^,^4^. However, the availability and feasibility of surgery as an option to all those who would potentially benefit, as well as its success, are far from optimal^5^,^6^. For example, sustained positive outcome twelve months post-surgery is only achieved in around one half of patients, and can be as low as 15% in extra-temporal cases (see refs 7,8, and references therein). Furthermore, many cases are evaluated but do not proceed to surgery because data fail to identify an optimal resection zone^9^. A challenge in identifying the cause of surgery failure results from the potential involvement of both short-term and longer-term mechanisms. However, cases with recurrence of seizures in the short-term are most likely to remain intractable^7^,^8^,^10^ and are presumed to be the result of an inadequate surgical resection, such that seizure-generating networks still remain.

A prevalent theoretical concept underpinning the surgical treatment of epilepsy is that of the *epileptogenic zone*, i.e. an “area of cortex that is indispensable for the generation of epileptic seizures”^11^. The goal of epilepsy surgery in this paradigm is the removal or disconnection of the epileptogenic zone^11^, since in a “localised” view of epilepsy such perturbations would render a patient seizure free. Unfortunately, this is a retrospective definition; the epileptogenic zone can only be assumed to have been part of the brain tissue resected or to have been disconnected by the resection in patients for whom surgery was successful. It is not, therefore, a notion that aids the prediction of an optimal surgical strategy. Rather, a combination of observations is acquired during pre-surgical planning regarding aspects of the epileptic brain that are presumed to correlate with the epileptogenic zone. These include, for example, regions of the brain that are first to produce pathologic electrographic activity when seizures occur (*seizure onset zone*) and regions that display evidence of abnormal structure that may contribute to ictogenicity (*epileptogenic lesions*)^11^. Concordance of these features is presumed to indicate a clear epileptogenic zone and in these cases surgery is more likely to succeed^12^. However, non-concordance of these features often occurs^13^ and even in cases for which well-defined focal epileptogenic zones caused by specific lesions are assumed, (for example mesial temporal lobe epilepsy) surgical resection does not always lead to seizure freedom^2^,^7^,^10^,^14^.

Collectively, this evidence highlights that our current understanding of ictogenesis is insufficient in that it does not help us to understand or predict the effects of surgical intervention in many cases. A potential factor contributing to our lack of understanding is that surgery focuses on the properties of individual regions of brain tissue, with insufficient regards to the *networks* within which they function^15^,^16^,^17^,^18^,^19^,^20^,^21^. The term network refers to a collection of *nodes* and *edges* (connections between nodes, see ref. 19 for a recent, epilepsy focussed review). Networks are crucial for brain function at various spatial scales, from interactions between neurons within a small region of the brain to large-scale brain networks mediated by white matter axonal projections. When studying a particular (dys-)function, decisions are necessarily made regarding the scale of network of interest. This can be informed, for example, by the nature of data that is readily acquired for studying a particular problem, or the scale at which the phenomenon of interest exists. Since we focus here on the clinical setting, where the expression of seizures requires large regions of the brain^17^, and standard data acquisition techniques yield dynamics relating to large regions of brain tissue, we focus on networks in which the nodes are regions of brain tissue and edges represent data-inferred connections between these regions.

It is important to note that the emergent behaviour of a network (for example the emergence of spontaneous pathological dynamics) may not be easily predicted from a straightforward dissection of the contributions from intrinsic properties of the individual nodes and those attributed to the connections between them. This renders understanding seizure generation within a complex brain network a difficult task. However, we can utilise mathematical techniques arising from complexity theory to help elucidate the links between node dynamics, network connectivity and seizure generation^18^,^22^,^23^. For example, when placed in a dynamic framework, the connectivity structure of a network has been shown to have a plausible role in determining whether seizures appear focal or generalised, and changes in node properties or connections between nodes were equally able to generate both seizure types^18^. However, the concepts of network dynamics have thus far not been applied to study the success or failure of surgical interventions for epilepsy. It remains unclear, for example, why surgery might not work in some cases, how seizures can arise in non-lesional epilepsies, why results from different pre-surgical evaluation methods may appear non-concordant, and perhaps most crucially, how to use current and future evaluation methods constructively to predict an optimal surgical strategy.

Thus the goal of this paper is to introduce a predictive, network dynamics paradigm for epilepsy surgery. To achieve this, we introduce a mathematical modelling framework that allows the study of the relationship between network structure, node dynamics and generation of discharges. As part of this framework we quantify the ability of a network to generate emergent pathological dynamics (e.g. discharges or seizures), a quantity that we term *Brain Network Ictogenicity* (*BNI*)^24^. In the specific model presented, we use spiking as a proxy for discharges. Quantifying the ictogenicity of networks in this way also allows us to quantify the effect of perturbations to specific nodes of the network, thus uncovering the mechanistic contribution of each node to the emergent ictogenicity, which we term *Node Ictogenicity* (*NI*). These definitions further enable us to evaluate the performance of potential surgical strategies *in silico*. We first demonstrate the use of these methods in exemplar networks, highlighting counter-intuitive facets of the emergence of pathological dynamics that include ictogenicity in the absence of intrinsically pathological nodes, widely distributed ictogenic mechanisms and non-trivial correspondence between the location of ictogenic nodes and the observed distribution of pathological dynamics.

In order to demonstrate the potential use of this framework in practice, we apply our methods to data derived from patients who underwent epilepsy surgery, and for whom post-operative outcome in terms of seizure control was measured. We derive functional networks from patient electrocorticogram (ECoG) recordings and quantify the distribution of *NI* across these networks. We find that nodes predicted to have large *NI* were more likely to lie within tissue that was subsequently resected when patient outcome was good (Engel class I or II) than when no improvement post-surgery was achieved (Engel class IV, see ref. 12 for a description of the Engel classification scheme). As a further test of model predictions, we use the model to calculate the expected reduction in *BNI* given the surgery that was performed (which we term Δ*BNI*). We find Δ*BNI* to be higher for patients with good outcome compared to patients with poor outcome, thus providing a preliminary validation of model predictions. Furthermore, we demonstrate the successful use of Δ*BNI* as a prognostic marker for post-operative outcome.

## Results

### Mathematical model of ictogenesis in brain networks

Here we provide quantitative definitions with which to study ictogenesis in networks. We start with a connectivity structure, and place a mathematical model onto each node that can generate recurrent transitions into pathological dynamics. Subsequently, we simulate this system in order to observe the emergent dynamics of the network and therefore determine the likelihood of emergent pathological dynamics. This allows us to quantify the extent to which a given network is ictogenic and quantitatively compare ictogenesis before and after specific perturbations are made, thereby assigning mechanistic contributions to nodes.

**Figure 1:**
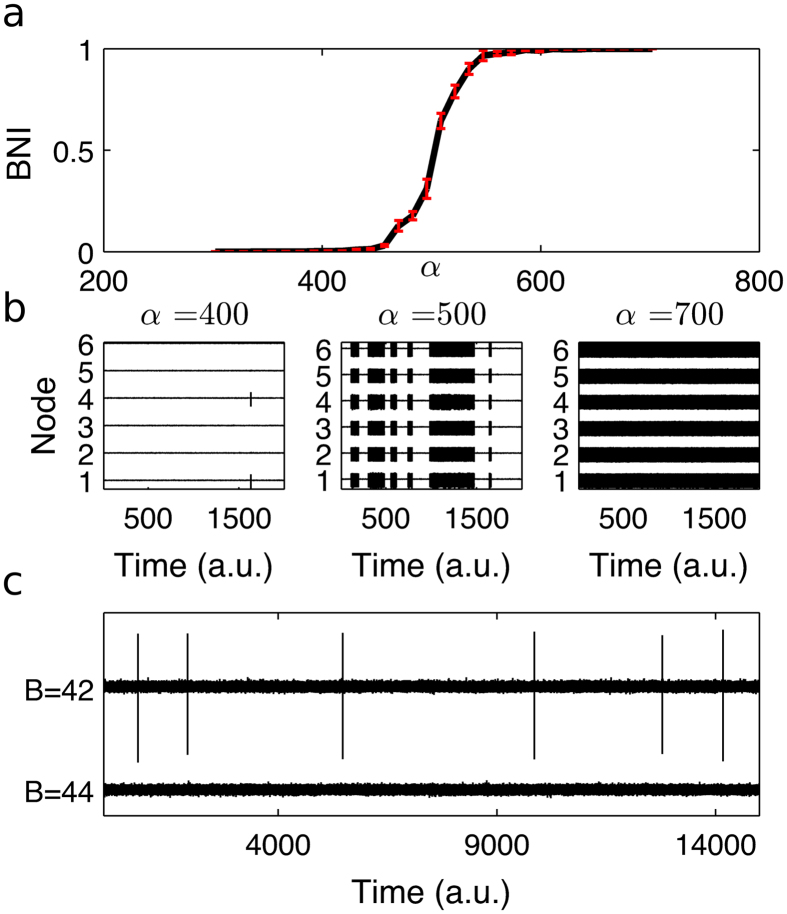
Model behaviour depends on network coupling strength and intrinsic node settings. (**a**) Increasing global coupling strength, α, leads to increases in *BNI*. The mean and standard error of *BNI* are shown for a single network at different values of α (see Fig. 3a(i) for the topology of this network). (**b**) Exemplar model dynamics for different values of α demonstrating that the model can generate either “healthy” or recurrent pathological dynamics, depending upon the magnitude of α. (**c**) Time series showing the output of a single node when the parameter *B* (relating to the strength of inhibitory feedback) is set to 42 (pathological node) or 44 (healthy node).

Since our aim is to study the emergence of pathological dynamics in networks, the crucial ingredients for such a model are its ability to be formulated in terms of networks and for it to generate transitions between “healthy” and “pathological” dynamic states. Consequently, several choices exist for the model to generate dynamics at the network nodes (see e.g.^25^,^26^,^27^,^28^,^29^,^30^). Here we choose to focus on neural mass models, which have been widely used in studies of brain dynamics in health and disease (see e.g.^31^,^32^). Such models have been shown to support the existence of dynamics similar to many epileptiform rhythms commonly observed clinically^22^,^25^,^33^ and provide a natural way to connect nodes (via long range, delayed synaptic connections)^34^. Neural mass models typically represent the dynamics of populations of excitatory and inhibitory neurons. They consist of linear impulse responses that model the rise and fall of population membrane potentials due to afferent activity and non-linear (sigmoidal) functions to convert net membrane potentials into efferent firing rates (see e.g.^26^ for a review). This approach has been used to model different circuits of the brain including thalamo-cortical loops, cortical microcircuits and distributed brain networks^31^,^34^,^35^. The specific neural mass model that we use embodies a circuit of interactions between principal neurons, excitatory interneurons, and inhibitory interneurons with two different time scales of inhibition^25^. The model has previously been used to generate an array of focal seizure dynamics^36^, and can generate repeated discharges due to noise driving^25^ (see Fig. 1). Model equations, parameters and further specifics are provided in the Methods section.

Mathematically, discharges in this model can arise due to different mechanisms, such as bistability, bifurcations, excitability or intermittency. We have previously identified parameters that place the model close to a saddle-node on invariant circle (SNIC) bifurcation^36^. In this regime, a model node can generate discharges due to noise (i.e. it is excitable, see Fig. 1), and this can propagate through networks. This configuration therefore allows to measure ictotenicity, as required. Figure 1 further demonstrates that the degree of “excitability” of the model, in the sense of how likely it is to generate discharges, depends upon the choice of parameter *B* (which embodies the strength of slow inhibitory connections). Based upon the analysis of 36, we choose a value of *B* = 44 for “normal” nodes and *B* = 42 for “pathological” (“hyper-excitable”) nodes.

### Quantification of brain network ictogenicity (*BNI*)

To study the extent to which a given network is ictogenic, we consider the enduring likelihood of a brain network to generate pathological dynamics, which we call *Brain Network Ictogenicity* (*BNI*)^24^. A practical approach for quantifying this is by summing the total time spent in discharges compared to a reference time period:


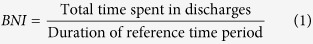


Figure 1 demonstrates that *BNI* is dependent upon the choice of global connectivity strength, α (see Methods and equations (4 and 5)). We therefore define a reference value of α for each network, which is the value such that the network spends approximately 50% of its time in pathological dynamics (i.e. *BNI* = 0.5). Although in reality most patients will spend significantly less than half of the time having seizures or discharges, our aim is to study the measurable effect that changes to the network have on *BNI*. Thus, the choice of 0.5 as a reference value facilitates detection of such changes in a computationally efficient manner.

### Quantification of node ictogenicity (*NI*)

We then study the contribution of each element of a network to ictogenesis. We define this contribution by the effect on *BNI* of perturbing that element of the network. In principle, we could envisage various perturbations (for example reflecting different possible treatments), but we focus here on the removal of nodes, which is unambiguously implemented in the model and has direct analogy with epilepsy surgery.

We therefore define “node ictogenicity” (*NI*) as the alteration to *BNI* that occurs upon removal of a node from the network. We are able to predict this effect in the model by setting all incoming and outgoing connections of a particular node to zero:


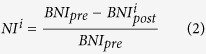


where *BNI*_*pre*_ is a reference state in which the unperturbed network generates a *BNI* of 0.5 (discharges 50% of the time: in practice this could be tuned using patient specific information) and 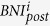 is the value of *BNI* after removal of node *i*. In some cases, *NI* may be negative, indicating an increase in *BNI* following the perturbation. In the current study we set such values to zero, as we consider them to represent non-beneficial effects. For each network, *NI* is calculated for each node, therefore providing a distribution of values.

We extend the definition of *NI* above to allow the removal of sets of nodes from a network, which is likely to be undertaken in surgery in practice. For a given set of nodes *n*_*s*_, with *n*_*s*_ = {*n*_1_, .., *n*_*M*_}, where M is the number of nodes to be removed (*M* > 1), Δ*BNI* is defined naturally as:


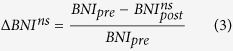


where 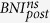 is the value of *BNI* after removal of all nodes in *n*_*s*_.

**Figure 2:**
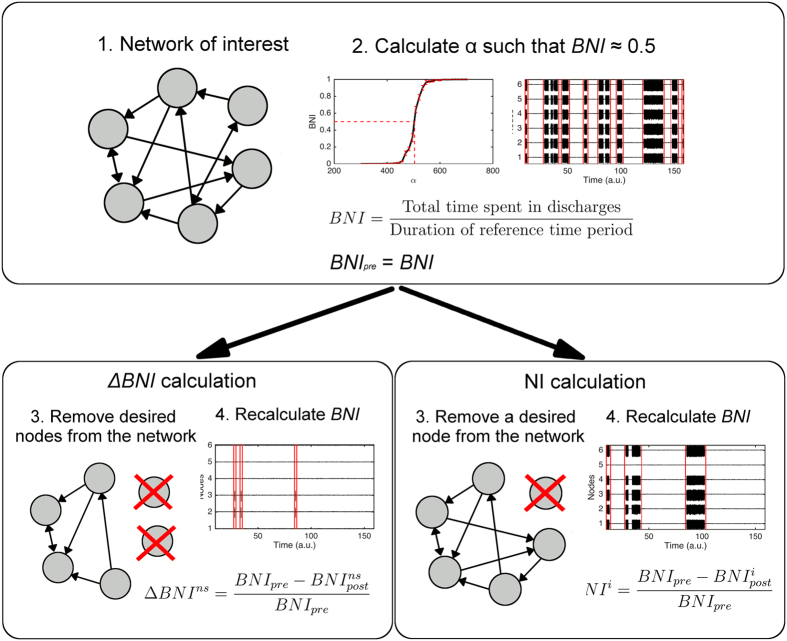
Schematic representation of the presented framework.

A schematic demonstrating the calculation of *BNI*, *NI* and Δ*BNI* is shown in Fig. 2.

### Emergent dynamics and node ictogenicity in exemplar networks

**Figure 3:**
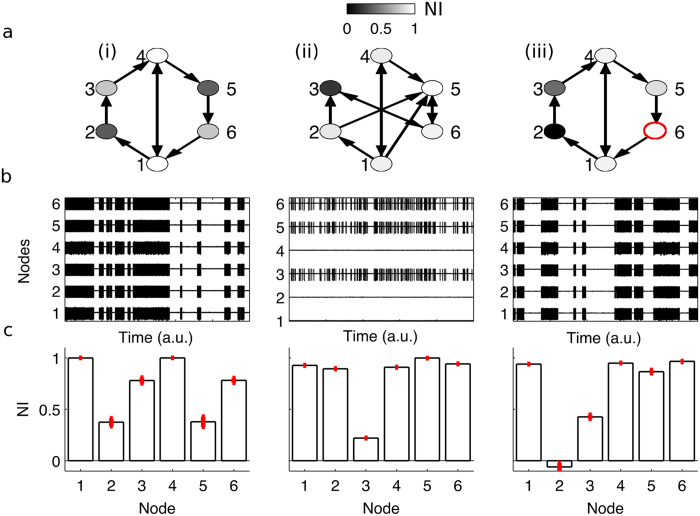
The distribution of node ictogenicity (*NI*) and resulting dynamics depends on network structure. (**a**) The distributions of *NI* are shown for three exemplar networks. Nodes are grey-scale coded according to their *NI*, with lighter colours indicating high *NI*. Arrows represent the presence and direction of connections. The red circle around node six of network (iii) indicates that this node is hyperexcitable (*B* = 42). (**b**) The dynamics of each network are shown, with signals labelled according to the network nodes. The dynamics of each node can be regarded as a single channel of simulated electroencephalogram (EEG)/ECoG, with low amplitude activity representing normal interictal activity, and bursts of high amplitude activity representing discharges. (**c**) The mean and standard error of *NI* for each node in each network (ten repeats per network).

The quantitative measures of network and node ictogenicity described above were applied to exemplar, six node networks (Fig. 3) in order to demonstrate their use and to explore counter-intuitive factors that might explain failure of current methods for defining surgical strategies. The choice of six node networks was made in order to strike a balance between computational tractability and the inclusion of a sufficient number of nodes to allow complex network structures. However, we proceed in subsequent sections to study larger, patient networks and also provide an example of equivalent calculations performed on a ten node network in Supplementary Fig. 1.

Networks (i) and (ii) of Fig. 3 each have six, identical nodes, but different connectivity structures. Simulating their dynamics (Fig. 3b) reveals that the emergent patterns of discharges are qualitatively different, with network (i) exhibiting a generalised pattern of discharges (i.e. all nodes are involved) and network (ii) displaying focal dynamics (discharges being restricted to three of the six nodes). The arrangement of network edges is therefore sufficient to determine whether the network will generate focal or generalised discharges; in some networks, focal discharges will be generated without the presence of an inherently abnormal node^18^.

Here, we extend beyond the observation of dynamics of networks and quantify the contribution to emergent ictogenicity of each node, by calculating *NI*. In Fig. 3a, *NI* is indicated by the shading of nodes in the network representation, with white nodes contributing more to ictogenicity than black nodes. Quantitative values of *NI* are provided in Fig. 3c. Figure 3 demonstrates that in addition to determining the dynamics of epileptiform activity, changes to connectivity structures affect how ictogenicity is distributed throughout a network. Network (i), for example, has two nodes that stand out as contributing significantly to discharge generation, whereas five out of six nodes contribute significantly to the ictogenicity of network (ii). Comparing the dynamics of networks (i) and (ii) in Fig. 3b highlights that relationships between observed dynamics (i.e. discharge patterns as observed with electroencephalogram (EEG) or ECoG) and the underlying ictogenic mechanisms can be counter-intuitive. Network (i) has “focal” mechanisms, but generalised dynamics and *vice versa* for network (ii). Furthermore, Fig. 3 demonstrates that whether a node is involved in pathological electrographic activity may not be sufficient to determine its involvement in ictogenesis. This is perhaps most striking in network (ii), which has three nodes that do not display emergent discharges but contribute significantly to ictogenicity (nodes one, two and four).

However, it is not only structure that contributes to emergent pathological dynamics. There is an interplay between the intrinsic dynamics of nodes and the connections between them. In network (iii) of Fig. 3 the structure of edges is identical to that of network (i), but at node six of network (iii) we place an inherently hyper-excitable pathology (by setting *B* = 42, see Fig. 1). We find that despite now having a network with a single hyper-excitable node, the ictogenic mechanisms become *more* distributed throughout the network (c.f. network (i)). In attempting to understand this effect, we note that the structure of edges in networks (i) and (iii) means that nodes one and four receive and supply more connections than other nodes. Hence, we might expect them to be more important for the generation of discharges. However, in network (iii), the hyper-excitability of node six makes it a driver of ictogenesis. Therefore, the *cycle* of edges connecting nodes one, four, five and six becomes crucial for discharge generation and hence all nodes within this cycle are more ictogenic than nodes two and three in network (iii).

### Quantification of patient NI distributions

The previous section highlights potential difficulties that are faced when using observations of signals recorded from different brain regions alone to make predictions for surgical interventions. These difficulties arise due to non-trivial relationships between the emergence of dynamics on networks, and the distribution over many nodes of contributions to ictogenesis. However, the quantitative framework we propose offers the possibility to inform predictions as it is based on methods that account for the interactions between components of an individual’s ictogenic network, and the emergence of pathological dynamics. We therefore sought to test the potential applicability of our method in sixteen patients who had undergone epilepsy surgery. The post-surgical outcome of each patient was classified according to the Engel scheme (Engel I: free of disabling seizures; Engel II: rare disabling seizures (“almost seizure-free”); Engel III: worthwhile improvement; Engel IV: no worthwhile improvement). Specific patient details are provided in Supplementary Table 1.

In order to apply this approach to patients in practice, the estimation of a relevant ictogenic network is required, given patient data. Since ECoG is commonly used in pre-surgical evaluation, we define a functional connectivity network based upon ECoG recordings^37^. Henceforth, each ECoG channel is referred to as a node and functional connections between these nodes (calculated using surrogate-corrected mutual information^37^) are referred to as edges. Further details regarding the derivation of patient networks are provided in the Methods section.

**Figure 4:**
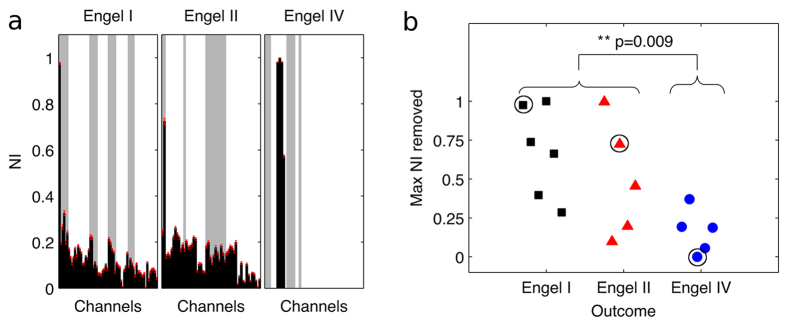
Comparison between model predictions and location of resections. (**a**) Exemplar *NI* distributions (black bars) are shown for three patients with different post-surgical outcome. Black bars indicate mean *NI* values for each node and red lines indicate their standard error (over ten repeats of calculations of *NI* with different realisations of noise). Grey bars indicate channels that were located above resected brain tissue. (**b**) Maximum *NI* of nodes that were included in the resection, grouped by post-surgical outcome. Braces indicate a Wilcoxon rank-sum test of differences in maximum *NI* between good (classes I and II, n = 11) and poor (class IV, n = 5) responders. Black circles around three of the markers highlight the patients presented in (**a**).

The distributions of *NI* within these functional networks were calculated for all sixteen patients. Exemplar profiles are given in Fig. 4a, which demonstrates that typical distributions of *NI* are skewed, with a small number of nodes having large *NI*. In this context, nodes with largest *NI* are the nodes that the model predicts are particularly crucial for ictogenesis and at least one such node should be resected. We therefore analysed the overlap of resected tissue with nodes having largest *NI* (Fig. 4b). Patients with good response to surgery (outcome classes I or II) displayed significantly higher values of largest *NI* in resected tissue than patients with poor outcome (class IV) (Fig. 4b, Wilcoxon rank-sum test, p < 0.01).

**Figure 5:**
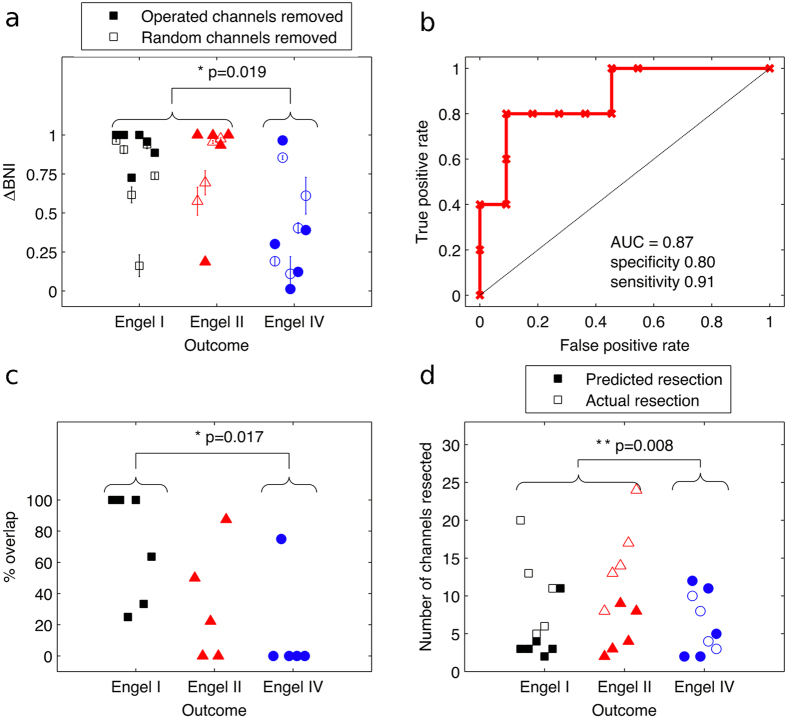
Quantification of model predictions for sixteen patients. (**a**) Distribution of Δ*BNI* based on nodes that correspond to resected tissue (filled markers) or randomly selected channels (unfilled markers, mean and standard error over ten repeats). Random selections contained the same number of nodes as the actual resection. Braces indicate a Wilcoxon rank-sum test of differences in Δ*BNI* (operated nodes removed) between good and poor responders. (**b**) Receiver operating characteristic (ROC) analysis for good versus poor responders, using Δ*BNI* as a predictive measure. (**c**) Percentage overlap between model-predicted resections and actual resections, grouped by response class. Braces indicate a Wilcoxon rank-sum test of differences in % overlap between class I and class IV responders (**d**) Comparison of predicted resection size (filled markers) versus actual resection size (unfilled markers). Braces indicate a Wilcoxon rank-sum test between good and poor responders based on the difference between predicted and actual resection sizes. In the main text we refer to patients as ordered from one to sixteen based on their offset on the horizontal axis in (**a**,**c**,**d**).

Typically, and in the case of patients studied herein, resections involve the removal of tissue corresponding to multiple ECoG channels (i.e. multiple nodes). Thus it is of interest to consider a measure of *NI* for a subset of nodes that have been removed, rather than focussing on a single node. Therefore Δ*BNI* was calculated for the set *n*_*s*_ of resected nodes. Figure 5a demonstrates that the majority of patients with good outcome (classes I and II) have large values of Δ*BNI*, whereas the majority of patients with poor outcome (class IV) have lower values. The median value of Δ*BNI* was found to be significantly larger for patients in outcome classes I and II than for outcome class IV (Wilcoxon rank-sum test, p < 0.05, Fig. 5a). This represents a validation of model predictions in patient data, since Δ*BNI* is the *in silico*, quantitative prediction for the reduction in ictogenicity of the suggested surgery. For comparison, calculations of the effect of removing random sets of nodes from the network were also performed. Interestingly, three out of five patients in class IV have smaller Δ*BNI* for their actual resection than for resections composed of randomly selected channels. This indicates that alternative resection strategies can be found in the model.

To test the potential use of these predictions for prognosis at the individual level, a receiver operating characteristic (ROC) analysis using Δ*BNI* as a classifier was performed and revealed a sensitivity of 0.91 and specificity of 0.8 for classifying good (class I or II) versus poor (class IV) outcome (Fig. 5b). Patients eight (class II) and thirteen (class IV) (numbers according to ordering on the x-axis of Fig. 5a) are notable outliers that lead to a less than perfect classification. Future work will aim to ascertain the reasons for the presence of these outliers.

A further advantage of the approach outlined herein is the ability to make predictions for optimal resections based upon the underlying mechanisms of ictogenesis (i.e. based upon individual patient networks). In order to test this possibility, model-based predictions for surgical resections were produced by rank-ordering the nodes of each patients’ networks according to their *NI*. The model was then used to calculate Δ*BNI* for simulated resections of increasing size, starting with the largest *NI* and sequentially adding nodes with lower *NI* to the resection. For each of the sixteen patients studied, this procedure resulted in a simulated resection that rendered the model incapable of generating discharges (i.e. having Δ*BNI* > 0.99, Fig. 5c,d). The resulting, patient-specific, predicted surgical strategies were compared to the resections that were actually performed by calculating the percentage overlap of channels in the predicted and actual resections (Fig. 5c). The median overlap between predicted and actual strategies was found to be significantly different among the different responder classes (Kruskal-Wallis test, p < 0.05). Pairwise comparisons revealed that the overlap was higher for Engel class I responders than Engel class IV responders (Wilcoxon rank-sum test, p < 0.05). This demonstrates an enhanced alignment of the surgery that was performed with model-predicted strategies in patients who are post-operatively seizure free.

A natural question that arises is whether the model-derived, predicted resection strategy would offer the additional benefit of being smaller (i.e. removing fewer nodes from the network) than the actual surgery performed. It was found that for all twelve patients with good outcome (classes I or II), predicted resections are smaller than or equal to performed resections. Three out of the five patients with poor outcome, however, have predicted resections that are larger than the performed resections. In order to quantify this effect, the difference in size between predicted and performed resections was calculated for each patient. This quantity differs significantly between patients with good (class I and II) and poor (class IV) outcome (Wilcoxon rank-sum test, p < 0.01), being more negative for good responders. Thus, the framework we present can be used to predict alternative surgical strategies with less extensive resections in most cases, but suggests larger resections (if possible) could potentially have been beneficial to some patients with poor post-operative outcome.

## Discussion

Although brain network phenomena are becoming recognised as important to fully understand ictogenesis, the additional challenges and clinical opportunities that this paradigm shift brings are not yet widely appreciated. In particular, a predictive modelling framework to quantify the contribution of network components to emergent ictogenesis has been lacking. Here, we sought to advance the way data regarding brain networks is used in the clinical setting by introducing tools to classify the contribution of nodes to ictogenesis in large-scale brain networks. The basis of our approach is the use of mathematical models to simulate recurrent pathological dynamics and methods by which to quantify global (whole brain) and local (brain regions) ictogenicity. Using these tools we were able to show how the ictogenicity of nodes can be quantified and provided concrete examples of the counter-intuitive relationships between network structure and inherently pathological nodes that can underlie ictogenesis and lead to the emergence of pathological dynamics (Fig. 3). Building on this framework, we subsequently applied the same method to examine the ictogenicity in networks inferred from preoperative ECoG data of epilepsy patients who had undergone resective surgery and whose post-operative seizure outcome was known. We found that patients with good post-operative outcome had undergone resections that the model predicted would lead to greater reduction in *BNI* than patients with poor post-operative outcome (Fig. 5). We subsequently used the model to predict resections that would significantly reduce *BNI* and found that alternative, smaller resections would achieve an equivalent effect for patients with good outcome. In contrast, model predictions of optimal resections for patients with poor outcome were not in keeping with the surgery actually undertaken; the model predicted a better outcome would have followed if different regions of tissue had been resected, and in some cases, more tissue.

The aim of uncovering qualitative or quantitative features of electrographic recordings that correlate with the epileptogenic zone is long-standing 11,38,39,40. Features that have shown particular promise are the presence of high frequency oscillations (HFOs)^38^,^39^ or the relationship between HFOs and other rhythms^40^. Reference 40 recently demonstrated that the percentage of tissue resected that contains phase-locked high gamma activity could classify good versus poor responders to surgery with an area under ROC curve (AUC) of 0.79. In our study, classification based on Δ*BNI* yielded an AUC of 0.87 (Fig. 5b), with only two patients accounting for any misclassification (patient 8, with Engel II and patient 13, with Engel IV). The accuracy of this novel classification method should now be tested in larger cohorts of patients.

Existing approaches to identify “ictogenic” tissue from ECoG signals typically involve uni- and multi-variate time series analyses^41^,^42^,^43^,^44^,^45^,^46^. Our framework has several potential advantages over and above these existing approaches. Perhaps most importantly, our use of mathematical models allows for the estimation of ictogenicity based on patient-specific information (i.e.network structures and properties of nodes). This in turn allows quantitative comparisons to be made amongst alternative, individualised, surgical strategies. There are many potential benefits to this, including the possibility to widen the availability of surgery to patients for whom traditional approaches find non-concordance of imaging or overlaps between putative surgical targets and important (eloquent) brain tissue^9^. In the latter case, our framework allows alternative resection strategies to be tested *in silico*, with the strategy showing largest Δ*BNI* providing an “optimal” approach. Furthermore, our model-based approach allows for the integration of network and node-specific properties in making predictions regarding epilepsy surgery. If we assume the presence or particular properties of HFOs to be indicative of enhanced local “excitability” or ictogenicity (for example underpinned by altered firing patterns)^47^, such information can be directly incorporated into the model (Figs 1 and 3). Integration of information from different imaging modalities, for example combining features of scalp EEG, MEG, ECoG, fMRI and structural MRI is therefore also a possibility.

The patient networks studied herein were functional networks derived from ECoG electrodes during the first half of seizures. In future it will be important to test this approach using networks derived from different modalities, including anatomically derived networks, as well as functional and effective connectivity networks from different brain states^48^. Structural connectivity constrains the dynamics of the brain^49^, and is therefore often (erroneously) considered a “ground truth” for connectivity based large-scale brain modelling. A problem with this approach is that the repertoire of changes that modulate whether structural connections are currently “open” (in terms of the exchange of activity between regions of the brain)^50^ are not incorporated in most models. Rather, the “open” or “active” communication pathways of the brain are perhaps captured well by functional connectivity analysis, which could have enabled us to observe a link between model predictions based on functional connectivity and patient outcome. Future studies should address which kinds of networks contain most information regarding ictogenicity.

In the present study we employed node removal to classify ictogenic mechanisms. This has a clear analogy with epilepsy surgery, where the traditional aim is to resect nodes from the network. In principle, the framework we present can be extended to study other treatment perturbations, such as the removal of edges (analogous to the partial disconnection of a region of tissue)^51^ or the alteration of intrinsic node parameters (such as the widespread or localised effect of pharmacological or stimulus therapies). Other lesioning techniques such as radiofrequency thermocoagulation^52^ could be integrated with our approach since the same electrodes are used for both recording and therapeutic interventions.

By quantifying the contribution of nodes to emergent pathological dynamics, we demonstrated the highly paradoxical and non-intuitive finding that nodes that are seemingly uninvolved in seizures are potentially important for ictogenesis. This finding is in line with other emerging evidence that recordings from brain regions outside the “seizure onset zone” contain information regarding impending seizures^53^. Unfortunately, this means that observations of pathological dynamics on EEG or ECoG recordings (e.g. seizure onset zones or seizure spreading) may be insufficient to classify regions of tissue as important for seizure generation (e.g. epileptogenic zones), thus potentially contributing to the failure of surgical treatment in certain cases of intractable epilepsy^7^.

The findings we present suggest several areas for future development. Interestingly, our approach was able to distinguish between good and poor responders, but was not able to elucidate a significant difference between patients with Engel I and Engel II outcome. Although both of these classes indicate significant positive results post-surgery, it would be useful to advance our methods such that a more detailed prognosis can be offered. Analysis of larger patient cohorts and further studies of network dynamics under different choices of model may facilitate this advance. Several choices of mechanisms to describe the transition between healthy and pathological dynamics have been proposed, including noise-driven bistability^29^,^54^,^55^, intermittency^22^,^56^ and self-organised transients in networks of pulse-coupled oscillators^28^,^30^. Further improvements may be obtained through appropriately tuning the underlying model and network inference methods. In future studies we will seek to validate our approach in experimental models and larger patient cohorts.

In summary, we presented and validated a novel, model-based approach to quantify the ictogenicity of brain networks. Our approach allows causal relationships between properties of brain regions, network connectivity and the generation of pathological dynamics to be studied, thereby facilitating quantitative evaluation of different surgical strategies *in silico*. The framework we introduced therefore has the potential to advance the selection of optimal resections for patients undergoing epilepsy surgery.

## Methods

### Model

The model we use comprises the following set of ordinary differential equations (for further details see)^25^,^36^:


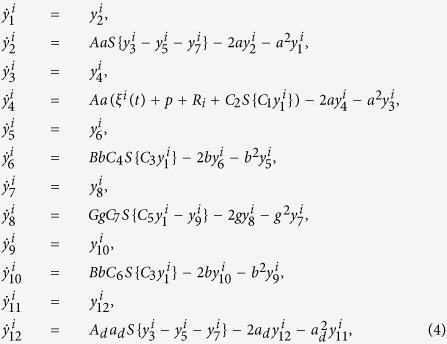


These twelve equations represent six post-synaptic potential (PSP) responses. PSPs are modelled as linear impulse responses, given by second order differential equations. Sequential pairs of variables (e.g. *y*_1_ and *y*_2_) represent the two-dimensional expansion of these equations, with PSP dynamics given by the first of these variables (i.e. *y*_1_, *y*_3_, *y*_5_, *y*_7_, *y*_9_, *y*_11_). Specifically, *y*_1_ is the PSP response of interneurons, *y*_3_ is the EPSP of pyramidal cells, *y*_5_ is the slow IPSP of pyramidal cells, *y*_7_ is the fast IPSP of pyramidal cells, *y*_9_ represents inhibition of the fast inhibitory population and *y*_1_1 represents a network propagation delay. Further details can be found in ref. 25. *R*_*i*_, represents input from other nodes of the network, via the binary adjacency matrix, D (with entries *d*_*kl*_), and is given by:


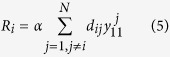


α is a parameter that globally scales the coupling strength and *N* is the number of nodes in the network. *S* represents a sigmoid nonlinearity common to neural mass models as follows:


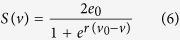


We introduce stochastic fluctuations into the model in the form of Gaussian noise, ξ^*i*^(*t*), that is independent across nodes and has variance σ^2^ (following)^25^. These equations were solved using an Euler-Murayama scheme, with step size 0.001 s and random initial conditions drawn from a normal distribution (N(0, 1)). Parameters of the model are given in Table 1.

Discharges in the model can be defined by the presence of large-amplitude spiking in contrast to low amplitude dynamics. The presence of spikes is easily determined by the amplitude of the model output in comparison to periods of time where non-spiking dynamics occur (see e.g. Fig. 1). For the purpose of tractability, a return from spiking to rest was deemed to have occurred if a quiescent period of two seconds (of model time) occurred following a spike. This period of time is sufficient to encompass the full trajectory of a spike based on visual inspection of the model dynamics of a single node. Spikes were extracted for each node by applying a threshold to the average absolute amplitude of the model output over a sliding window of length 0.05 s.

Given our choice of intrinsic node parameters and connectivity structure, free parameters remaining are the global scaling of connectivity strength, α, and the variance of white noise added to each node, σ^2^. We fixed σ^2^ = 3.41, a value chosen to ensure the generation of recurrent discharges.

### Quantification of *BNI* and *NI*

α was chosen such that *BNI* = 0.5, see Figs 1 and 2. α was estimated by first fixing a noise realisation and initial conditions, then using a root-finding algorithm to calculate the value of α for which BIN −0.5 was equal to zero. We performed this procedure ten times (for different instances of noise and initial conditions) and the median of these ten values was assigned to α for subsequent simulations.

To calculate *NI* (equation (2)), the model was simulated for 5,000 time points for a given noise realisation and *BNI*_*pre*_ calculated. Connections to and from node *i* were then set to zero, the simulation repeated with the same noise realisation, and *BNI*_*post*_ calculated. The estimation of *NI* was repeated ten times for each network and each node. Figure 3 demonstrates that this number of repetitions gives reliable estimations for *NI*.

### Patients and EEG acquisition

We analysed the EEG data set of 37 consisting of intracranial recordings of sixteen patients. The data were acquired during presurgical workup at the Inselspital Bern and the analysed multichannel EEG signals contained the first seizure that occurred during video-monitoring, including at least three minutes before seizure onset as well as after seizure termination. The average number of channels free of permanent artifacts was 60 (range 32–100). Eleven patients were female and median age was 31.0 years (range 19–59 years). Following resective therapy six patients fell into Engel class I (free of disabling seizures), five into Engel class II (rare disabling seizures) and five into Engel class IV (no worthwhile improvement). Median post-surgical follow up was 3.0 years (range 1 to 5 years). Further patient details are provided in Supplementary Table 1.

For data acquisition AdTech electrodes (Wisconsin, USA) and a NicoletOneTM recording system (VIASYS Healthcare Inc., Wisconsin, USA) were used. Before analysis EEG signals were down-sampled to a sampling rate of 512 Hz, re-referenced against the median of all the channels free of permanent artefacts as judged by visual inspection by an experienced epileptologist (K.S.) and digitally band-pass filtered between 0.5 and 150 Hz using a fourth-order Butterworth filter. All EEG recordings were carried out prior to and independently from our retrospective analysis of the data. Importantly, these recordings are not experimental but were recorded for clinical diagnostics and treatment of each individual patient only. In accordance with approved guidelines, the EEG recordings and additional information (age, localization of seizure onset, etiology and postsurgical outcome) was anonymized prior to our analysis. In addition, all patients had given written informed consent that their data from long-term EEG might be used for research purposes.

### ECoG analysis and coregistration

High resolution T1-weighted MRI was acquired before and after resective therapy in all patients. The MRIs were coregistered by affine transformations and the extent of the resection was determined by manual segmentation. After electrode implantation computed tomography (CT) was acquired to exclude bleeding. The metal contacts of the intracranial EEG electrodes were clearly visible as high intensity artifacts. Coregistration of the CT to the MR images allowed to determine the monitored brain regions with high resolution and its relation to the resected brain tissue. Full details of these procedures are described in ref. 37.

To extract functional connectivity matrices from the multichannel EEG signals we followed the procedures described in ref. 57. Briefly, 10 sets of multivariate iterative amplitude adjusted Fourier transform (IAAFT) surrogates were generated over moving data windows of 8 seconds length. The mutual information matrix was calculated from 10 partially overlapping segments of 2 seconds duration from the original and the surrogate data. This allowed to estimate the surrogate corrected mutual information measure introduced in ref. 57 by nonparametrically comparing the distribution of 10 values for the original time series to 100 values of the surrogates in each window. This method yielded time-varying functional connectivity networks, as previously analysed by^37^. Since we focus on the mechanisms leading to the generation (or onset) of discharges or seizures, and since functional networks derived from early in the progression of seizures have been shown to correlate well with epileptogenic tissue^37^, we focus our analysis on networks derived from the first half of seizures.

## Additional Information

**How to cite this article**: Goodfellow, M. *et al*. Estimation of brain network ictogenicity predicts outcome from epilepsy surgery. *Sci. Rep.* **6**, 29215; doi: 10.1038/srep29215 (2016).

## Supplementary Material

Supplementary Information

## Figures and Tables

**Table 1:** Parameter values and their meanings.

Parameter	Interpretation	Value
*A*	Mean excitatory synaptic gain	5 mV
*B*	Mean slow inhibitory synaptic gain	44 mV
*G*	Mean fast inhibitory synaptic gain	20 mV
*A*_*d*_	Gain of delayed efferent activity	3.25 mV
*a*	Inverse average time constant - excitatory feedback loop	100/s
*b*	Inverse average time constant - slow inhibitory feedback loop	50/s
*g*	Inverse average time constant - fast inhibitory feedback loop	500/s
*a*_*d*_	Inverse average time constant for delayed efferent activity	100/s
*C*_1_	Connectivity strength - pyramidal to excitatory	135
*C*_2_	Connectivity strength - excitatory to pyramidal	0.8*C*_1_
*C*_3_	Connectivity strength - pyramidal to slow inhibitory	0.25*C*_1_
*C*_4_	Connectivity strength - slow inhibitory to pyramidal	0.25*C*_1_
*C*_5_	Connectivity strength - pyramidal to fast inhibitory	0.3*C*_1_
*C*_6_	Connectivity strength - slow inhibitory to fast inhibitory	0.1*C*_1_
*C*_7_	Connectivity strength - fast inhibitory to pyramidal	0.25*C*_1_
*v*_0_	Firing threshold potential	6 mV
*e*_0_	Half of maximum firing rate of neural masses	2.5/s
*r*	Slope of potential to rate sigmoid function at *v* = *v*_0_	0.56/mV
*p*	Baseline input firing rate to pyramidal population	90/s

Model parameter values and biophysical interpretations^25^,^36^.
