# Supplementary material for: Estimation of brain network ictogenicity predicts outcome from epilepsy surgery
Source: Sci Rep. 2016 Jul 7;6:29215. doi: 10.1038/srep29215 (PMC4935897; doi:10.1038/srep29215)
Supplement: Supplementary Information [file srep29215-s1.pdf]

## Supplementary Information: Estimation of brain network ictogenicity predicts outcome from epilepsy surgery

Goodfellow, M, Rummel, C., Abela, E., Richardson, M.P., Schindler, K. and Terry, J.R.

Exemplar ten node network

In order to demonstrate the application of our methods to larger artificial networks, the distribution of NI for a ten node network is shown in Supplementary Figure S1. This larger network serves as an additional example of the potential lack of correspondence between the presence of epileptiform activity and high NI (compare Fig. 3B in the main text).

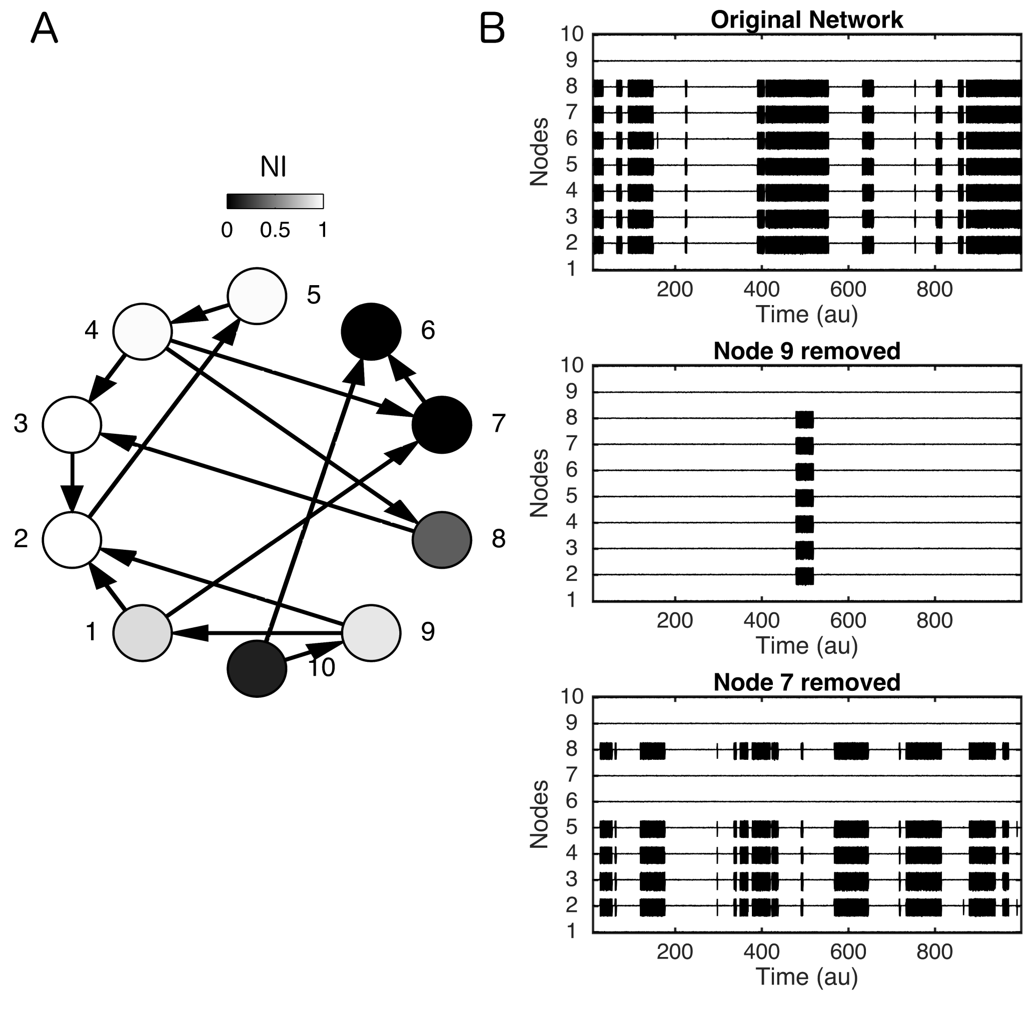


**Supplementary Figure S1**: Example of NI calculation in a ten node network. (A) Network structure with grey-scale coded NI. (B) Example model dynamics in the intact network (top) and upon removal of nodes 9 (middle) and 7 (bottom).

## Supplementary Table 1: Detailed patient information

| Patient | Engel class | Gender | Age (y) | Syn-drome | Hemi-sphere | Lesion MRI visible | Total No. of iEEG channels | No. of artifact free iEEG channels | No. of resected iEEG channels | Resection type | Follow up (y) |
|---|---|---|---|---|---|---|---|---|---|---|---|
| 1 | I | F | 26 | MTLE | R | y (hippocampal sclerosis) | 64 | 64 | 20 | amygdalo-hippocampectomy | 3 |
| 2 | I | F | 48 | MTLE | L | y (hippocampal sclerosis) | 64 | 64 | 13 | amygdalo-hippocampectomy | 3 |
| 3 | I | M | 27 | LTLE | L | n | 56 | 56 | 5 | temporo-lateral resection | 1 |
| 4 | I | M | 36 | PLE | L | y (pilocytic astrocytoma) | 77 | 74 | 6 | lesionectomy | 5 |
| 5 | I | F | 19 | MTLE | L | y (hippocampal sclerosis) | 44 | 40 | 11 | amygdalo-hippocampectomy | 5 |
| 6 | I | F | 25 | FLE/TLE | R | n | 104 | 99 | 11 | temporo-polar resection | 4 |
| 7 | II | F | 49 | FLE | R | y (focal cortical dysplasia) | 102 | 92 | 8 | lesionectomy | 4 |
| 8 | II | F | 46 | LTLE | R | n | 102 | 100 | 13 | amygdalo-hippocampektomie | 3 |
| 9 | II | M | 20 | LTLE | R | n | 79 | 54 | 14 | temporo-polar resection | 3 |
| 10 | II | M | 31 | LTLE | L | y (hippocampal sclerosis) | 74 | 59 | 17 | temporo-lateral | 3 |
| 11 | II | F | 24 | LTLE | L | n | 50 | 47 | 24 | temporo-polar resection | 3 |
| 12 | IV | F | 38 | LTLE | L | n | 62 | 59 | 2 | temporo-lateral resection | 4 |
| 13 | IV | F | 23 | LTLE | L | n | 63 | 61 | 10 | temporo-lateral resection | 2 |
| 14 | IV | F | 59 | MTLE | L | y (space occupying amygdala) | 52 | 49 | 8 | lesionectomy | 4 |
| 15 | IV | M | 32 | PLE | L | y (focal cortical dysplasia) | 98 | 96 | 4 | lesionectomy | 2 |
| 16 | IV | F | 31 | FLE | R | y (tuberous sclerosis) | 37 | 32 | 3 | lesionectomy | 2 |
